# Gut microbiome and metabolome profiling in coal workers’ pneumoconiosis: potential links to pulmonary function

**DOI:** 10.1128/spectrum.00049-24

**Published:** 2024-09-16

**Authors:** Xiao Yu, Tao Xiong, Lu Yu, Gaisheng Liu, Fan Yang, Xueqin Li, Yangyang Wei, Xiaojing Wang, Shuting Wei, Yi Jiang, Xiaomei Kong, Shouan Ren, Yiwei Shi

**Affiliations:** 1 NHC Key Laboratory of Pneumoconiosis, Shanxi Key Laboratory of Respiratory Diseases, Department of Pulmonary and Critical Care Medicine, The First Hospital of Shanxi Medical University, Taiyuan, China; 2 Quality Control Office, Xishan Occupational Disease Prevention and Control Institute, Taiyuan, China; 3 Department of Respiratory Medicine, Jincheng General Hospital, Shanxi, China; Tainan Hospital, Tainan, Taiwan, China

**Keywords:** CWP, 16S rRNA sequencing, gut microbiota, LC-MS, fecal metabolome, pulmonary function

## Abstract

Coal workers’ pneumoconiosis (CWP) is a severe occupational disease resulting from prolonged exposure to coal dust. However, its pathogenesis remains elusive, compounded by a lack of early detection markers and effective treatments. Although the impact of gut microbiota on lung diseases is acknowledged, its specific role in CWP is unclear. This study aims to explore changes in the gut microbiome and metabolome in CWP, while also assessing the correlation between gut microbes and alterations in lung function. Fecal specimens from 43 CWP patients and 48 dust-exposed workers (DEW) were examined using 16S rRNA gene sequencing for microbiota and liquid chromatography-mass spectrometry for metabolite profiling. We observed similar gut microbial α-diversity but significant differences in flora composition (β-diversity) between patients with CWP and the DEW group. After adjusting for age using multifactorial linear regression analysis (MaAsLin2), the distinct gut microbiome profile in CWP patients revealed an increased presence of pro-inflammatory microorganisms such as *Klebsiella* and *Haemophilus*. Furthermore, in CWP patients, alterations in gut microbiota—particularly reduced α-diversity and changes in microbial composition—were significantly correlated with impaired pulmonary function, a relationship not observed in DEW. This underscores the specific impact of gut microbiota on pulmonary health in individuals with CWP. Metabolomic analysis of fecal samples from CWP patients and DEW identified 218 differential metabolites between the two groups, with a predominant increase in metabolites in CWP patients, suggesting enhanced metabolic activity in CWP. Key altered metabolites included various lipids, amino acids, and organic compounds, with silibinin emerging as a potential biomarker. Kyoto Encyclopedia of Genes and Genomes pathway enrichment analysis linked these metabolites to pathways relevant to the development of pulmonary fibrosis. Additionally, studies on the interaction between microbiota and metabolites showed positive correlations between certain bacteria and increased metabolites in CWP, further elucidating the complex interplay in this disease state. Our findings suggest a potential contributory role of gut microbiota in CWP pathogenesis through metabolic regulation, with implications for diagnostic biomarkers and understanding disease mechanisms, warranting further molecular investigation.

**IMPORTANCE:**

The findings have significant implications for the early diagnosis and treatment of coal workers’ pneumoconiosis, highlighting the potential of gut microbiota as diagnostic biomarkers. They pave the way for new research into gut microbiota-based therapeutic strategies, potentially focusing on modifying gut microbiota to mitigate disease progression.

## INTRODUCTION

Coal workers’ pneumoconiosis (CWP) is a severe occupational disease characterized by diffuse pulmonary fibrosis resulting from prolonged inhalation of coal dust in the workplace. Despite decades of preventive measures, CWP remains a significant global public health challenge. From 1990 to 2017, the global incidence of pneumoconiosis surged from 36,186 to 60,055, marking a 66% increase, with CWP contributing proportions of 27% and 25%, respectively (1). This challenge is particularly acute in developing nations that rely heavily on coal as their primary energy source. In China and India, over 20 million workers are exposed to coal dust in their workplaces (2). In China, coal dust pneumoconiosis constitutes approximately 50% of newly reported cases in the annual diagnoses of pneumoconiosis. Despite widespread recognition, the disease’s pathogenesis remains poorly understood, with a notable absence of early diagnostic markers and specific therapies to impede disease progression (3). Compounding this issue is the hysteresis associated with coal workers’ pneumoconiosis, where the disease can manifest even after the cessation of dust exposure (4). Consequently, the prevalence of CWP is unlikely to decline soon, emphasizing the critical need to develop early diagnostic markers and more effective therapies to enhance our understanding of the disease’s molecular basis.

The gut–lung axis describes the close connection between the gut and the respiratory system, which provides new insights into the pathogenesis of a wide range of lung diseases (5). Despite their anatomical differences, the gut and lung share the same embryonic origin and mucosal structure, suggesting a unified immune system between them. Approximately 70% of the immune system is distributed across the intestinal barrier, and gut microbes are the primary defense system of this barrier (6). As a bridge to the gut–lung axis, gut microbes play a crucial role in regulating the respiratory immune response by modulating the immune system and producing short-chain fatty acids (SCFAs), among other things (7). Recent research has found that the gut microbiome of patients with chronic obstructive pulmonary disease (COPD) exhibits unique features accompanied by reduced levels of short-chain fatty acids compared to healthy individuals (8). In addition, a rapid decline in pulmonary function and the development of emphysema were observed in patients’ fecal transplantation experiments via mice. The change in the structure of the gut microbiota, as revealed through fecal transplantation experiments in mice, may play a causal role in the pathogenesis of COPD (9). Similarly, the gut microbiota of lung cancer patients is significantly different from that of healthy individuals, and specific gut microbes can be used for the potential prediction of early lung cancer (10). In addition, metabolites such as butyrate produced by the gut microbiota have been shown to prevent pneumonia by modulating gut flora and macrophages (11). In studies on pneumoconiosis, it has been observed that the gut microbiota of pneumoconiosis patients exhibits an increased abundance of the Prevotellaceae family compared to healthy individuals (12). Simultaneously, the abundances of nine other microbial species significantly decreased, indicating alterations in the microbial composition associated with this disease. Further animal research reveals time-dependent changes in the gut microbiota during exposure to silica (13). The time-specific interactions between the gut microbiota and metabolites may represent potential mechanisms underlying silica-induced lung injury. These research findings underscore the potential diagnostic and therapeutic value of studying the gut microbiota and metabolomic profiles in pneumoconiosis.

However, previous studies have generally compared healthy individuals as the control group, overlooking dust-exposed workers (DEW) with a high risk of disease but not yet diagnosed with CWP. Although both CWP patients and dust-exposed workers share a history of dust exposure and work in similar environments, their final outcomes are markedly different. Given this disparity, we hypothesize that changes in the gut microbiota may play a role in the pathogenesis of CWP. Therefore, by comparing the gut microbiota composition of these two groups, we can gain deeper insights into these differences. In this study, we chose DEW as the control group and employed 16S rRNA gene sequencing and untargeted metabolomics to comprehensively compare the differences in the gut microbiota and fecal metabolome of CWP patients. Our objective is to elucidate the pathogenic mechanisms of the disease through multiomics correlation analysis, with a specific focus on the impact of the gut microbiota on early lung function impairment in these patients.

## MATERIALS AND METHODS

### Study population

From January 2021 to October 2022, we conducted a study at the First Hospital of Shanxi Medical University, enrolling 43 patients diagnosed with CWP from a mining area in Taiyuan, Shanxi. Diagnoses of all patients with CWP were confirmed by a minimum of three occupational disease physicians in accordance with the “Diagnosis Criteria for Pneumoconiosis” (GBZ70-2015) and guidelines from the International Labour Organization. The diagnostic process involved verifying a reliable history of occupational exposure to coal dust, comparing X-ray examinations with diagnostic standard films for coal workers’ pneumoconiosis, and considering clinical manifestations and occupational health surveillance data. The diagnostic criteria for stage one CWP included one of the following manifestations: a small shadow with an overall density of grade 1, distributed in at least two lung zones, or exposure to asbestos dust resulting in a small shadow with an overall density of grade 1, distributed in only one lung zone, along with the presence of pleural plaques, or exposure to asbestos dust resulting in an overall density of 0 for small shadows, but with at least two lung zones having a density of 0/1, along with the presence of pleural plaques. We also recruited 48 dust workers from the same mining area as a control group. They have been exposed to coal dust for at least 5 years but have not exhibited symptoms of the disease, and their chest X-rays show normal lung conditions with no apparent visible abnormalities. The two groups were matched in terms of body mass index (BMI) and gender, and they both originated from the Taiyuan region of Shanxi, exhibiting relatively consistent dietary habits, which helped control confounding factors to a certain extent. The exclusion criteria for both groups included a history of lung cancer, other diffuse pulmonary fibrosis diseases, major respiratory system diseases in the previous year (such as chest trauma, lung surgery, pneumonia, pleurisy, emphysema, and asthma), recent antibiotic use within the past 3 months, and being under the age of 18. Demographic information for the enrolled participants was collected.

### Pulmonary function testing

The pulmonary function tests were performed by specialized doctors from the Pulmonary Function Unit at the First Hospital of Shanxi Medical University, using the Master Screen IOS instrument from Jaeger Co, Germany. To uphold the accuracy and reliability of the tests, all procedures strictly adhered to the American Thoracic Society/European Respiratory Society Pulmonary Function Test Standards (14). Considering that the study population is Chinese, we referred to relevant domestic literature to define normal pulmonary function ranges. According to these standards, normal pulmonary function is determined as follows: FEV1% pred ≥ 80%, FVC% pred ≥ 80%, and FEV1/VC% pred ≥92% of the predicted value (15, 16). In contrast, pulmonary function is classified as abnormal if any of the mentioned parameters fall below the defined thresholds. The predicted values utilized in the assessments are derived from data specific to the Chinese population, with adjustments made for gender, age, height, and weight to ensure precision in determining expected levels.

### Fecal sample collection and storage

The freshly collected feces of each participant were transported to the laboratory in ice bags as soon as possible and stored in a −80°C refrigerator.

### Fecal microbiota analysis

#### Genomic DNA extraction and PCR amplification

The genomic deoxyribonucleic acid (DNA) of the samples was extracted using cetyltrimethylammonium bromide. Then, the purity and concentration of the DNA were detected by agarose gel electrophoresis. An appropriate amount of the sample DNA was taken into a centrifuge tube and diluted with sterile water to 1 ng/µL. The diluted DNA was used as a template for polymerase chain reaction (PCR) amplification of the high variant region V3-V4 of the 16S rDNA using specific primers with barcodes (515F-806R), New England BioLabs Phusion High-Fidelity PCR Master Mix (with GC buffer) and high-efficiency high-fidelity enzymes.

#### PCR product mixing and purification

PCR products were detected by electrophoresis using agarose gel with 2% concentration; the PCR products qualified for detection were purified by magnetic beads, quantified by enzyme labeling, and mixed in equal quantities according to the concentration of PCR products. The PCR products were detected by electrophoresis using 2% agarose gel after sufficient mixing. The products were recovered using the gel recovery kits provided by Qiagen for the target bands.

#### Library preparation and sequencing

A TruSeq DNA PCR-Free Sample Preparation Kit (Illumina, USA) was used for library construction. Qubit and Q-PCR quantified the constructed library, and after the library was qualified, NovaSeq 6000 was used for online sequencing.

### Fecal metabolomic analysis

#### Metabolite extraction

One hundred microliters of the sample should be placed in an EP tube, to which 400 µL of 80% methanol in water should be added. The mixture was vortexed and shaken, allowed to stand in an ice bath for 5 minutes, and then centrifuged at 15,000 × *g* for 20 minutes at 4°C. A portion of the supernatant should be diluted with water suitable for mass spectrometry until the methanol content is 53%. The supernatant should then be collected and injected into the liquid chromatography–mass spectrometry (LC-MS) for analysis. All samples from which metabolites were extracted were processed in the same batch.

#### UHPLC-MS/MS analysis

Ultra-high performance liquid chromatography-tandem mass spectrometry (UHPLC-MS/MS) analysis was performed using a Vanquish ultrahigh-performance liquid chromatography system (Thermo Fisher, Germany) and an Orbitrap Q Exactive HF mass spectrometer (Thermo Fisher, Germany). The parameters of the chromatographic column (Hypersil Gold column, 100 × 2.1 mm, 1.9 µm, Thermo Fisher, USA) were set as follows: column temperature: 40°C; flow rate: 0.2 mL/min; positive mode: mobile phase A (0.1% formic acid) and mobile phase B (methanol); negative mode: mobile phase A (5 mM ammonium acetate, pH 9.0) and mobile phase B (methanol). The solvent gradient was set as follows: 2% B, 1.5 minutes; 2%–100% B, 12.0 minutes; 100% B, 14.0 minutes; 100%–2% B, 14.1 minutes; 2% B, 17 minutes. The mass spectrometer was operated in the positive/negative polarity mode with a spray voltage of 3.5 kV, capillary temperature of 320°C, sheath gas flow rate of 35 psi, aux gas flow rate of 10 L/min, S-lens RF level of 60, and aux gas heater temperature of 350°C.

### Gut microbiome data analysis

#### Paired-end read assembly and quality control

Paired-end reads were assigned to samples based on their unique barcodes and truncated by clipping barcodes and primer sequences. The paired-end reads were merged using FLASH to obtain the raw tags. Raw tags are quality-filtered under specific filtering conditions according to the QIIME quality control process to obtain high-quality clean tags. The tags are compared to a reference database (SILVA138.1 database) (17) using Vsearch to detect chimeric sequences, and then, the chimeric sequences are removed. Finally, effective tags are obtained.

#### OTU cluster and species annotation

The Uparse algorithm was utilized to cluster all effective tags of all samples, and by default, the sequences were clustered into operational taxonomic units (OTUs) with 97% consistency. The sequences with the highest number of occurrences were selected as the representative sequences of OTUs according to the principle of the algorithm. According to the Mothur (Version 3.8.31) (18) algorithm, the Silva database was used to annotate the classification information for each representative sequence. Multiple sequence comparisons were performed using MUSCLE software to investigate the phylogenetic relationships of different OTUs and the differences between dominant species in different groups. Finally, the data of each sample were homogenized. The data with the least amount of data in the sample were used as the criterion for homogenization. The subsequent α- and β-diversity analyses were based on the homogenized data.

#### Alpha-diversity

Four indices were calculated using QIIME to analyze the α-diversity of the sample species. The Chao1 and ACE indices were selected to identify community richness, and the Shannon and Simpson indices were selected to identify community diversity. Intergroup differences in α-diversity indices were analyzed using the Wilcoxon rank-sum test and displayed using R software.

#### Beta-diversity

β-Diversity analyses were used to assess differences in species complexity among samples, and QIIME software calculated Bray‒Curtis distances between samples of the microbial populations. Nonmetric multidimensional scaling diagrams (NMDS) were plotted using the Vegetarian Package in R software and tested for the significance of differences in the community structure between groups in conjunction with Adonis analysis.

#### Differential flora analysis

The Wilcoxon rank-sum test was performed on gut microbiota with a relative abundance greater than 1% at the phylum, family, and genus levels to determine species with significant differences between the CWP patients and DEW groups (*P*-value <0.05). Further analysis was performed using linear discriminant analysis (LDA) effect size (LEfSe) to detect significantly different colonies. Colonies were considered significantly different when the *P*-value <0.05 and the log LDA score ≥3.5. To gain more insight into microbiome association with CWP, we performed multivariate linear regression analysis (MaAsLin2) adjusting for age (19). Correction of *P*-values was performed by the Benjamini and Hochberg false discovery rate method, and only taxa with a *P*-value <0.05 and *Q*-value <0.05 were considered statistically significant.

#### Random forest analysis

Random forest is a supervised machine learning method. In this paper, we analyzed and modeled random forests for genus-level species abundance using the randomForest package in R software. MeanDecreaseAccuracy and MeanDecreaseGin screened important species, and then, each model was cross-validated (default 10-fold), and receiver operating characteristic (ROC) curves were plotted.

### Metabolomics analysis

#### Data processing and metabolite identification

Raw data files were imported into Compound Discoverer 3.1 library search software for processing. Simple filtering was performed for each metabolite’s retention time, mass-to-charge ratio, and other parameters. The retention time bias was set to 0.2 minutes, and the mass bias was set to 5 ppm to allow consistent peaks across samples for more accurate identification. Subsequently, the mass bias was set to 5 ppm, the signal intensity bias to 30%, the signal-to-noise ratio to 3, the minimum signal intensity, and the sum of ions extracted from the peaks. Molecular formulae were predicted based on molecular ion peaks and fragment ions and compared to the mzCloud, mzVault, and Masslist databases. Background ions were removed using blank samples, and raw quantification results were normalized to yield final metabolite identification and relative quantification results.

#### Metabolite annotation and differential metabolite screening

The identified metabolites were annotated using the Kyoto Encyclopedia of Genes and Genomes (KEGG), Human Metabolome Database, and LIPIDMaps databases. In multivariate statistical analyses, data were transformed using MetaX followed by partial least squares discriminant analysis (PLS-DA). In univariate analysis, the statistical significance (*P*-value) of each metabolite between the two groups was calculated based on the *t*-test. The metabolite levels’ fold change (FC) value between the two groups was calculated. Metabolites with variable importance in the projection (VIP) >1.0, *P*-value <0.05, FC >1.5, or FC <0.667 were considered differentially abundant metabolites. Volcano plots were created using the R software package (ggplot2), which integrates VIP values, log2 (fold change), and log10 (*P*-value) to visualize the overall distribution of differentially abundant metabolites.

#### KEGG enrichment analysis

The KEGG database was used to study the functions and metabolic pathways of these metabolites. Moreover, metabolic pathway enrichment of differentially abundant metabolites was performed. The metabolic pathway was considered enriched when *x*/*n* > *y*/*N* was satisfied. Additionally, when the *P*-value of a metabolic pathway was <0.05, the metabolic pathway was considered to be significantly enriched.

### Statistical analysis

Statistical analysis was conducted using SPSS 26.0. *t*-tests were applied for baseline data with a normal distribution, while the Wilcoxon rank-sum tests were employed for baseline data not conforming to a normal distribution. Categorized data were analyzed using χ^2^ tests. Correlations between differential flora and pulmonary function parameters and between different genera and metabolites were evaluated through Spearman correlation analysis. Significance levels were set at *P* < 0.05 (*), *P* < 0.01 (**), and *P* < 0.001 (***), indicating significant, highly significant, and extremely highly significant differences, respectively.

## RESULTS

### Participant information

The study included 43 male CWP patients and 48 male DEWs. Table 1 illustrates the demographics and occupational details of the participants. Significant differences emerged between the two groups regarding age and duration of employment, while BMI and smoking habits showed no significant variance. On average, the CWP patients were older and exposed to occupational dust longer than the DEW group. Although the two groups showed no significant difference in overall pulmonary function status, specific pulmonary function indices such as FEV_1_, FEV_1_/FVC%, FEV_1_/FVC% pred, MEF50% pred, and MMEF% pred were significantly lower in the CWP group compared to the DEW group.

**TABLE 1:** Descriptive analysis of participant characteristics in CWP patients (case group) and DEW (control group)

	CWP (*n* = 43)	DEW (*n* = 48)	*P*-value^*a*^
Age	60.00 (55.00, 65.50)	49.50 (45.00, 51.25)	**<0.001**
Duration of exposure (years)	32.00 (26.50, 35.00)	20.50 (17.00, 26.50)	**<0.001**
BMI	25.46 (23.32, 27.78)	25.88 (23.66, 27.88)	0.625
Smoking (year)	20.00 (0.00, 35.00)	21.50 (9.75, 28.00)	0.364
Smoking state, *n* (%)			0.205
No	14 (32.56)	10 (20.83)	
Yes	29 (67.44)	38 (79.17)	
Pulmonary function status, *n* (%)			0.205
Impaired	14 (32.56)	10 (20.83)	
Normal	29 (67.44)	38 (79.17)	
FEV_1_	2.87 ± 0.57	3.23 ± 0.56	**0.003**
FEV_1_% pred	93.57 ± 15.12	96.23 ± 15.69	0.413
FVC	3.83 ± 0.70	4.10 ± 0.64	0.055
FVC% pred	99.22 ± 13.70	99.93 ± 14.68	0.812
FEV_1_/FVC%	75.47 (72.30, 78.30)	78.96 (75.56, 82.11)	**<0.001**
FEV_1_/FVC% pred	90.60 (86.50, 93.64)	94.33 (90.16, 97.84)	**<0.001**
FEV_1_/VC%	74.46 (70.99, 76.53)	78.27 (73.21, 80.84)	**0.001**
FEV_1_/VC% pred	95.65 ± 7.56	98.23 ± 7.36	0.103
MEF25% pred	43.96 (36.62, 64.69)	59.93 (42.17, 70.62)	0.061
MEF50% pred	69.72 ± 23.35	80.15 ± 24.83	**0.042**
MMEF% pred	62.40 ± 20.93	72.03 ± 23.72	**0.044**
DLCO_CSB_% pred	93.20 (83.50, 105.80)	94.40 (88.30, 100.82)	0.833
DLCO/VA% pred	104.00 (96.40, 117.10)	103.60 (95.83, 110.78)	0.625

^*a*^ The bolded p-values indicate statistically signifcant results.

### Altered ecological diversity in the gut of CWP patients compared to DEW

**Fig 1:**
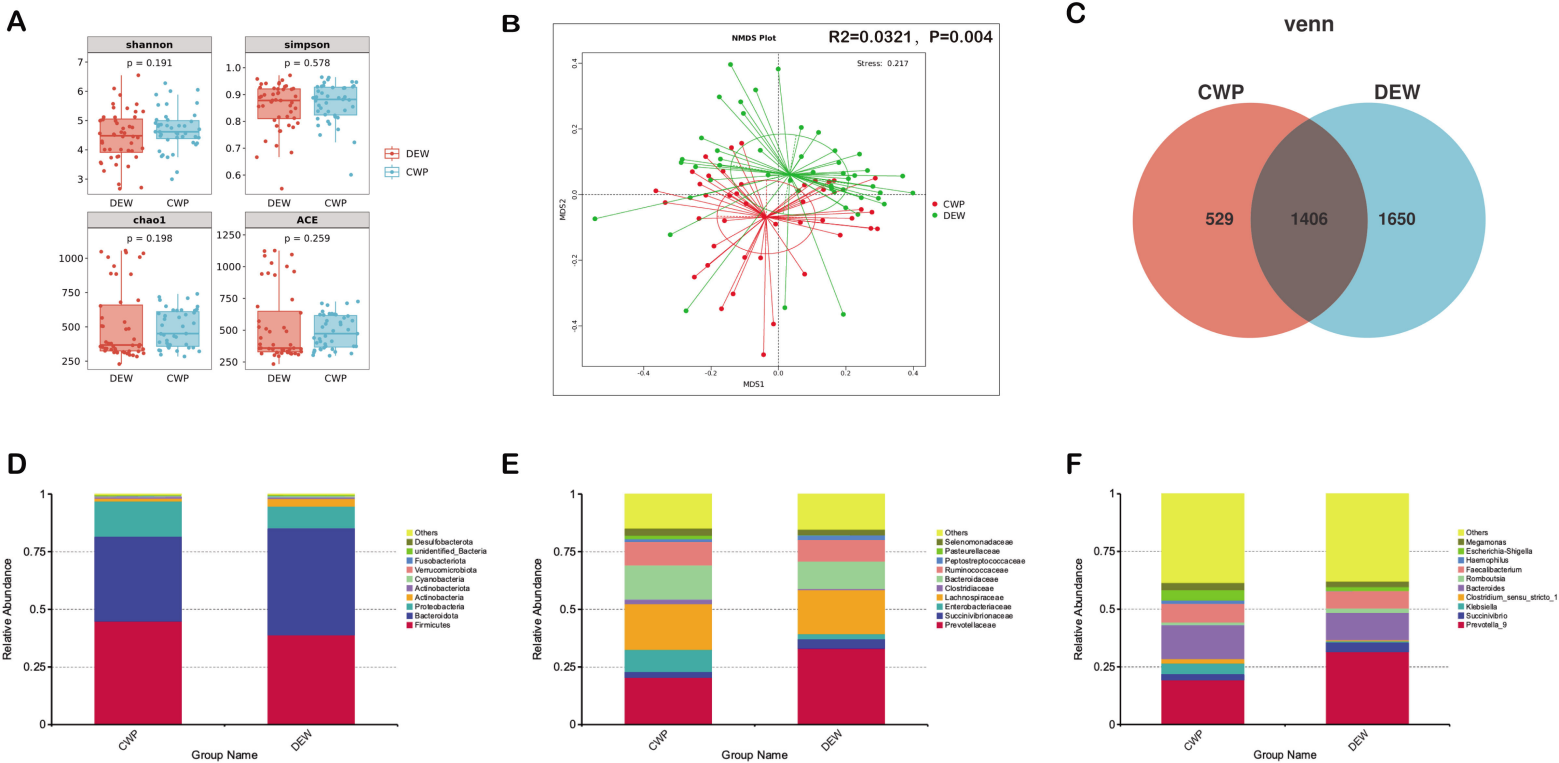
Gut microbiota composition altered in CWP patients. (**A**) Comparison of gut microbiota α-diversity. Similar α-diversity in CWP patients and DEW (by Shannon index, Simpson index, ACE index, Chao1 index, *P* > 0.05). (**B**) NMDS based on Bray‒Curtis distance. CWP patients and DEW divided into two clusters with significant differences in gut microbiota composition (for Bray‒Curtis distance, *P* = 0.004). (**C**) Venn diagram showing overlapping and specific OTUs between CWP patients and DEW. (**D**) The relative abundances of gut microbiota at the phylum level. (**E**) The relative abundances of gut microbiota at the family level. (**F**) The relative abundances of gut microbiota at the genus level.

The α-diversity of the gut microbiota in CWP patients and DEW was analyzed, revealing no significant differences in community richness and diversity (Fig. 1A). Subsequently, β-diversity was assessed between the two groups. NMDS analysis demonstrated a notable separation of samples between the two groups and a significant difference in the composition of the community structure (R2 = 0.0321, *P* = 0.004, Fig. 1B), with 529 OTUs unique to the patients, 1,650 OTUs unique to the workers, and 1,406 OTUs common to both groups (Fig. 1C). Histogram analysis of the top 10 most abundant species at the phylum, family, and genus levels revealed significant differences in the gut microbiota composition between CWP patients and DEW (Fig. 1D through F). Although CWP and DEW exhibited comparable gut microbial community richness and diversity levels, their compositions varied widely.

### Abnormal gut microbiota composition in CWP patients compared to DEW

The microbial composition between the CWP patients and the DEW group exhibited significant differences at several taxonomic levels (Table S1). Among the 30 identified bacterial phyla, Firmicutes, Bacteroidota, Proteobacteria, and Actinobacteria were the top four phyla in terms of abundance, accounting for 98.21% and 98.05% of the mean number of sequenced reads in the CWP and DEW groups, respectively. At the phylum level, no differential bacterial phylum was found between the two groups (*Q* > 0.05). At the family level, Clostridiaceae, Oscillospiraceae, Enterobacteriaceae, and Pasteurellaceae were significantly more abundant in CWP patients, while Peptostreptococcaceae, Veillonellaceae, and Sutterellaceae were significantly lower (*Q* < 0.05). At the genus level, we observed a significant reduction in the abundance of the anti-inflammatory microorganisms *Agathobacter* and *Romboutsia* in CWP patients (20, 21). In contrast, the abundance of the pro-inflammatory microorganisms *Clostridium_sensu_stricto*_1 (22), *Klebsiella* (23), *Escherichia-Shigella* (24), and *Haemophilus* (25) was significantly increased in CWP patients. Additionally, a rise in *Lachnospiraceae*_NK4A136_group, known for potential immune enhancement, was observed in CWP patients (26).

**Fig 2:**
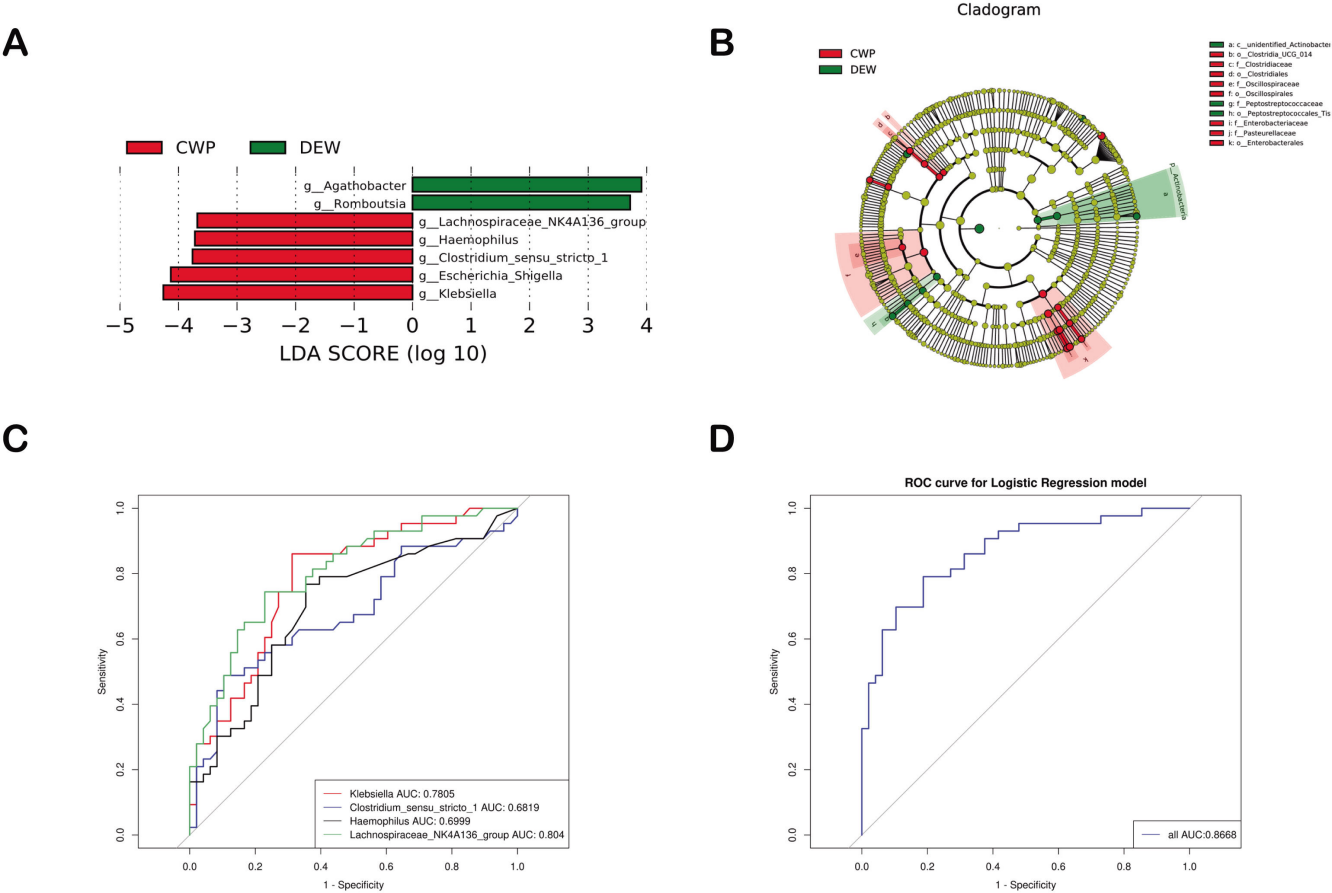
Differential gut microbiota analysis. (**A**). Differential taxa identified by LEfSe analysis (LDA > 3.5, *P* < 0.05). (**B**) Cladogram of bacterial taxa with significant differences in abundance; circles radiating from the inside out represent taxonomic levels from phylum to genus (or species). (**C**) Models with individual genus marker. (**D**) Model with combined genus marker. AUC, area under the curve.

The LeEfSe method was employed to identify groups of differential classifications at each taxonomic level. Seven different genera were found between the CWP and DEW groups at the genus level, consistent with the results of the above analysis (Fig. 2A and B). Considering the differences in age between the two groups and adjusting for age using MaAsLin2, genera such as *Klebsiella* (*Q* < 0.001), *Haemophilus* (*Q* < 0.421), *Clostridium_sensu_stricto*_1 (*Q* < 0.497), and *Lachnospiraceae*_NK4A136_group (*Q* < 0.001) remained distinct. These genera could potentially serve as disease markers for CWP (Fig. 2C and D).

### Predictive Modeling of CWP patient Microbiota

**Fig 3:**
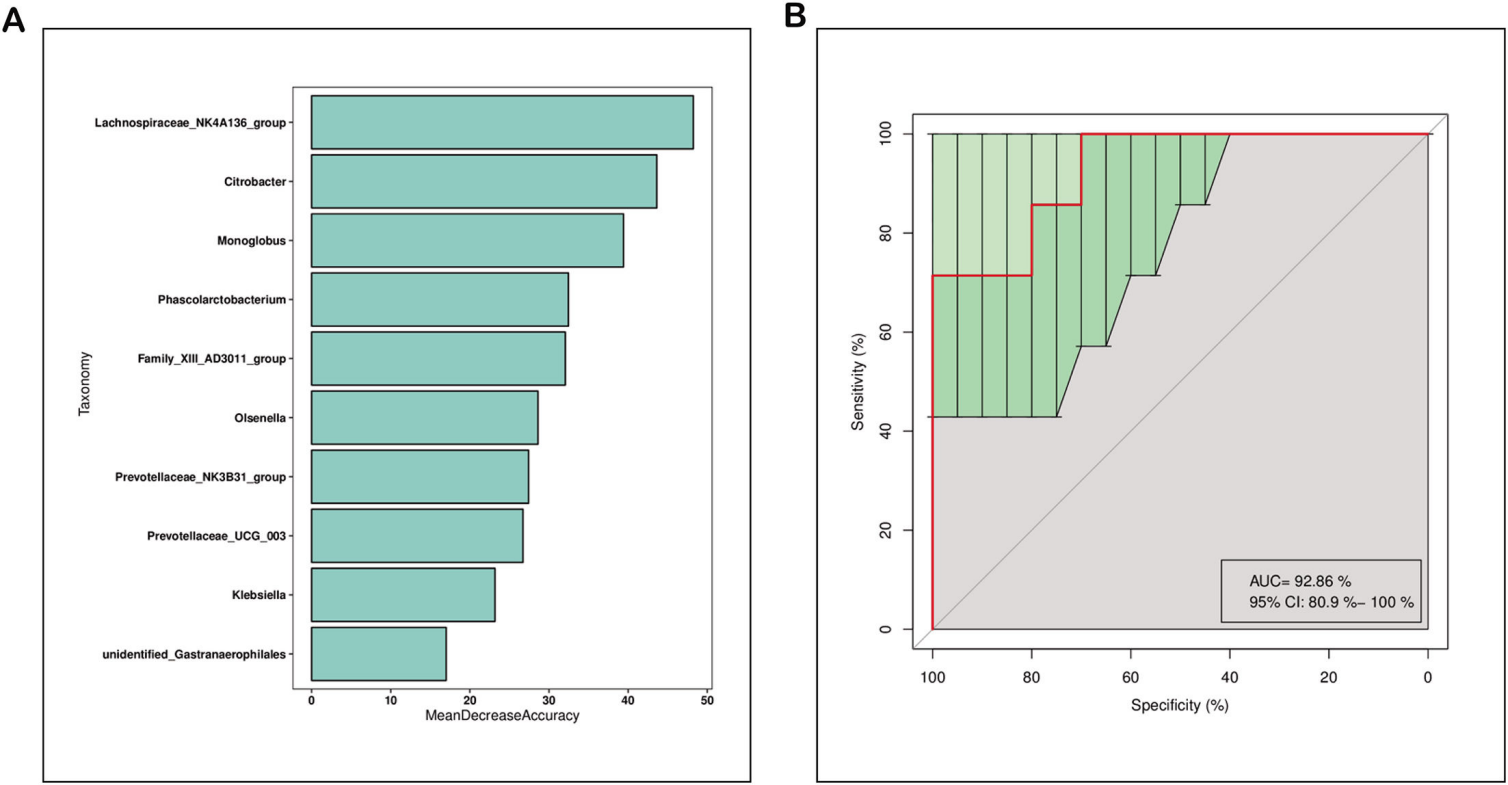
Prediction model of the airway microbiota for CWP status based on the genus-level relative abundances using random forests. (**A**) Variable importance ranking chart. MeanDecreaseAccuracy measures the degree to which a random forest’s prediction accuracy decreases by changing a variable’s value to a random number. A higher value indicates more importance. (**B**) ROC curve of the CWP model using 10 discriminatory genera.

A random forest-derived model employing microbial genera successfully differentiated CWP patients from DEW. This model exhibited a high AUC-ROC of 92.86% [95% confidence interval (CI): 80.90%–100%], suggesting that these genera are effective diagnostic markers (Fig. 3A and B). Notably, *Lachnospiraceae*_NK4A136_group was identified as having the highest diagnostic value, affirming the findings from LEfSe analysis.

### Gut microbiota characteristics in CWP patients with varied pulmonary function

**Fig 4:**
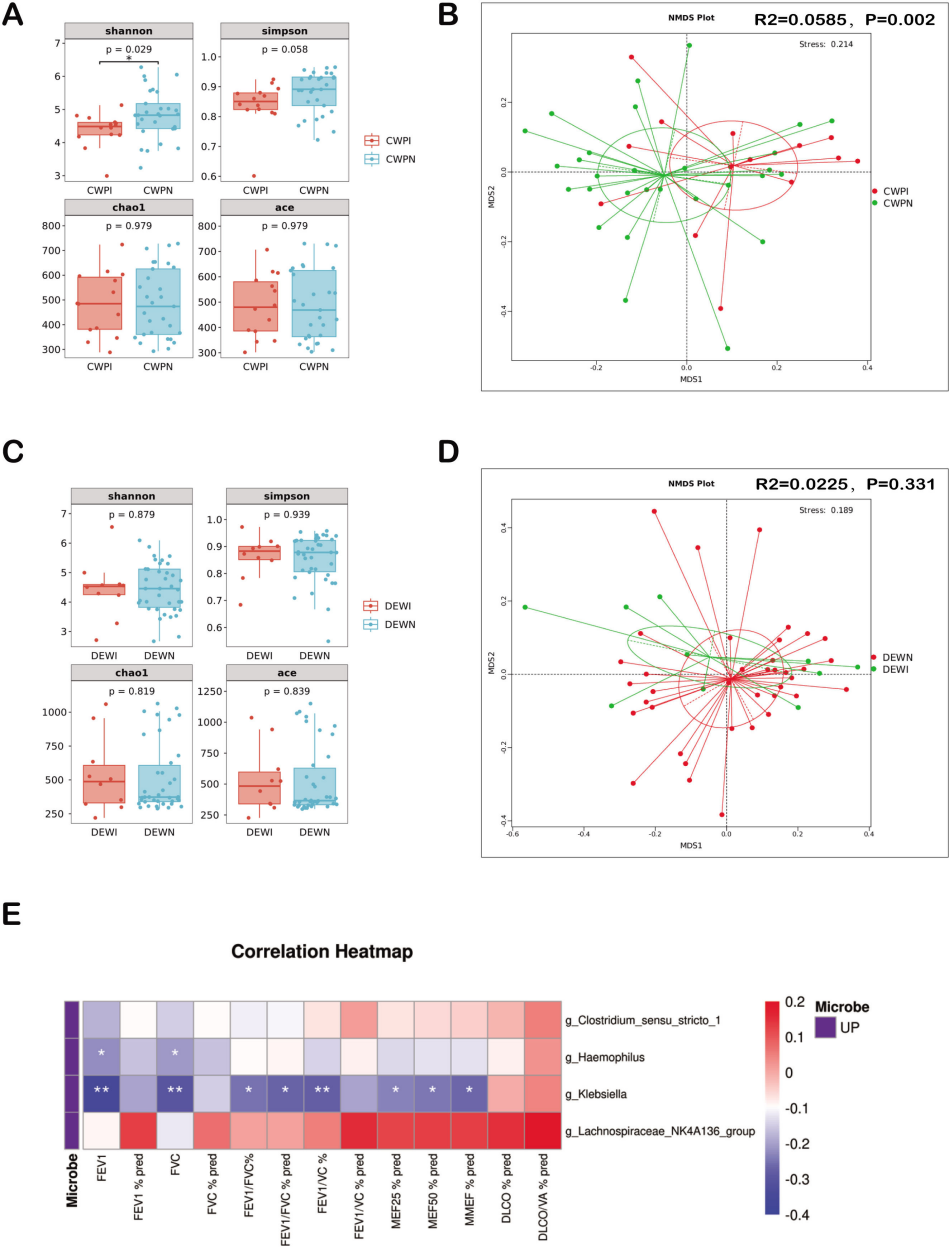
Gut microbes in CWP patients associated with decreased pulmonary function. (**A**) Analysis of microbial α-diversity in CWP patients with different pulmonary function statuses. Microbial α-diversity was lower in the group with impaired pulmonary function (CWPI) than in the group with normal pulmonary function (CWPN) (by Shannon index, *P* < 0.05). (**B**) Analysis of microbial β-diversity in CWP patients with different pulmonary function statuses. Significant differences in the gut microbiota composition between the two groups (Adonis for Bray‒Curtis distance, *P* = 0.002). (**C**) Analysis of microbial α-diversity in DEW with different pulmonary function statuses. Gut microbial α-diversity was similar in the group with normal pulmonary function (DEWN) and the group with impaired pulmonary function (DEWI). (**D**) Analysis of microbial β-diversity in DEW with different pulmonary function statuses. There were no differences in the gut microbiota composition between the two groups. (**E**) Spearman’s correlation between differential gut microbiota and pulmonary function parameters. Red indicates a positive correlation, while blue indicates a negative correlation. ^*^P-value <0.05, ^**^P-value <0.01, and ^***^P-value <0.001.

To gain insight into whether changes in gut microbiota impact pulmonary function, we compared the gut microbiota in CWP patients with normal and impaired pulmonary function. We observed significantly lower gut microbiota α-diversity (Shannon index) in CWP patients with impaired pulmonary function (Fig. 4A), accompanied by significant compositional changes (Fig. 4B). Multivariate linear regression models were used to adjust for the effects of age, BMI, and smoking status. The results showed that in patients with CWP, Shannon’s index was positively correlated with FEV_1_ (β = 9.25, *P* = 0.007) and FEV_1_/FVC (β = 5.26, *P* < 0.001), and Simpson’s index was positively correlated with FEV_1_ (β = 77.75, *P* = 0.012) and FEV_1_/FVC (β = 48.47, *P* < 0.001). Interestingly, these results were not observed in the DEW group(Fig. 4C and D). This discrepancy suggests that gut microbiota variations could particularly impact pulmonary function in CWP patients.

The correlation between differential gut flora and pulmonary function was further analyzed using Spearman correlation. The results showed that *Klebsiella* was significantly negatively correlated with various pulmonary function indices, whereas *Haemophilus* was also significantly negatively correlated with FEV_1_ and FVC. The potential negative impact of alterations in specific microbial communities on the lung health of CWP patients was emphasized.

### Metabolomic variances between CWP patients and DEW

**Fig 5:**
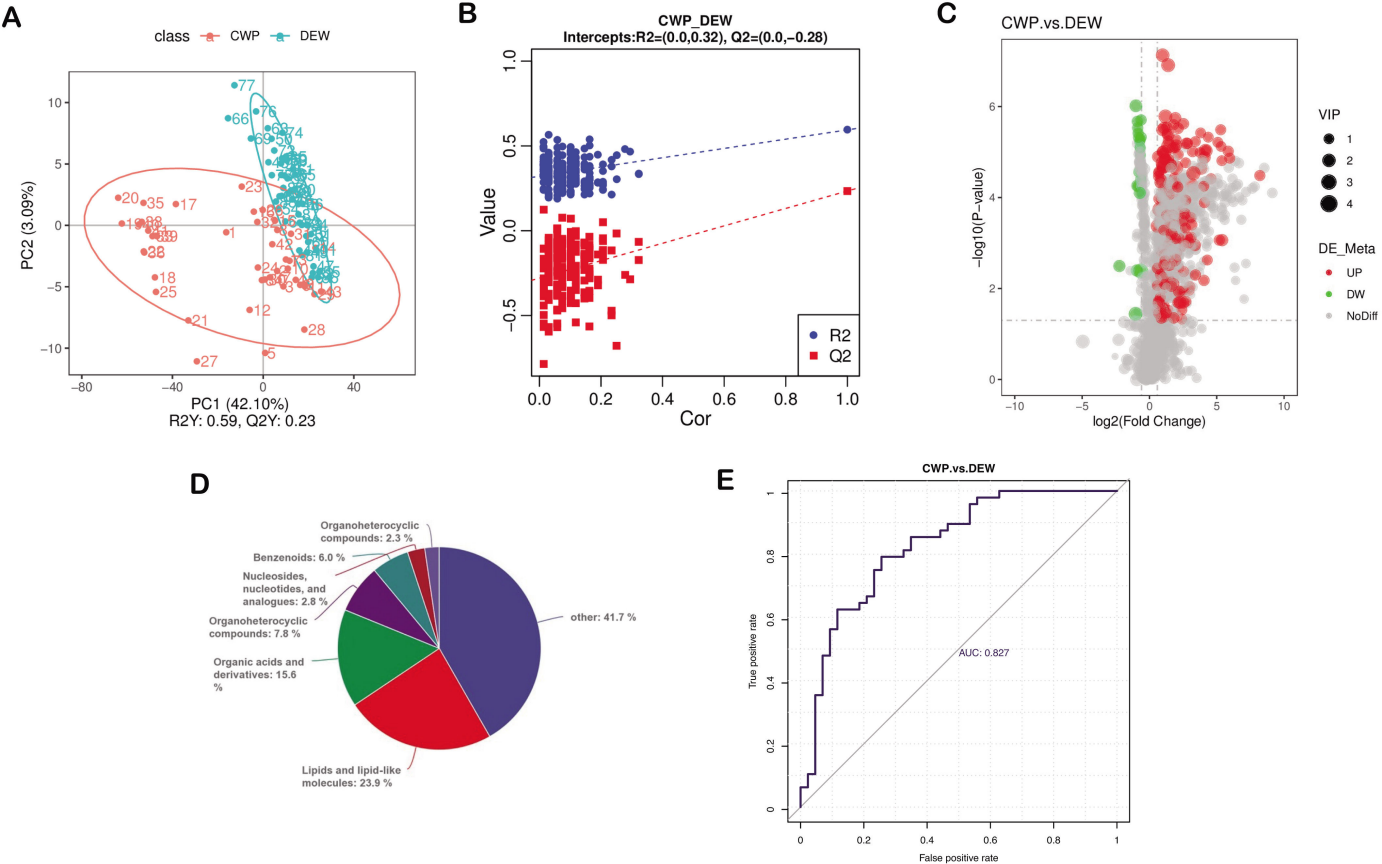
The metabolome of CWP patients differs from that of DEW. (**A**) PLS-DA shows that patients with CWP and DEW are divided into two different clusters. (**B**) The test for the PLS-DA model showed that the PLS-DA model for this study was valid. (**C**). Each point in the volcanic map represents a metabolite, with significantly upregulated metabolites represented by red dots and significantly downregulated metabolites represented by green dots. (**D**) Differentially abundant metabolite classification. (**E**) Generation of predictive ROC curves for the metabolite silibinin.

Nontargeted metabolomic analysis of fecal samples from CWP patients and DEW identified 1,416 metabolites. The PLS-DA score plot (Fig. 5A) illustrated a distinct separation between the two groups, while a negative *Q*^2^ intercept from the permutation test validated the reliability of the Orthogonal Partial Least Squares Discriminant Analysis (OPLS-DA) model (Fig. 5B). Based on VIP values >1 in the loading plot, FC ≥ 1.5 or FC ≤ 0.667, and *P* < 0.05, 218 differential metabolites were identified (Table S2). Only 21 (10.38%) of CWP patients showed a decrease (Fig. 5C), indicating that the CWP state may enhance metabolic activity. One hundred thirty-eight metabolites were categorized into groups, including various lipid types, amino acids and derivatives, and other organic compounds (Fig. 5D). We focused on these categories since lipids and amino acids represented 58.70% of the identified metabolites. Elevated levels of certain glycerophospholipids, amino acids, arachidonic acids, carnitine, and organic oxides were notable in CWP patients. Conversely, certain glycerophospholipids, flavonoid lignans, and long-chain fatty acids were decreased. Silibinin emerged as a significant potential biomarker with a high predictive value (AUC = 0.827), as indicated by the ROC curve analysis for metabolite prediction (Fig. 5E).

### Enrichment of metabolic pathways in CWP

**Fig 6:**
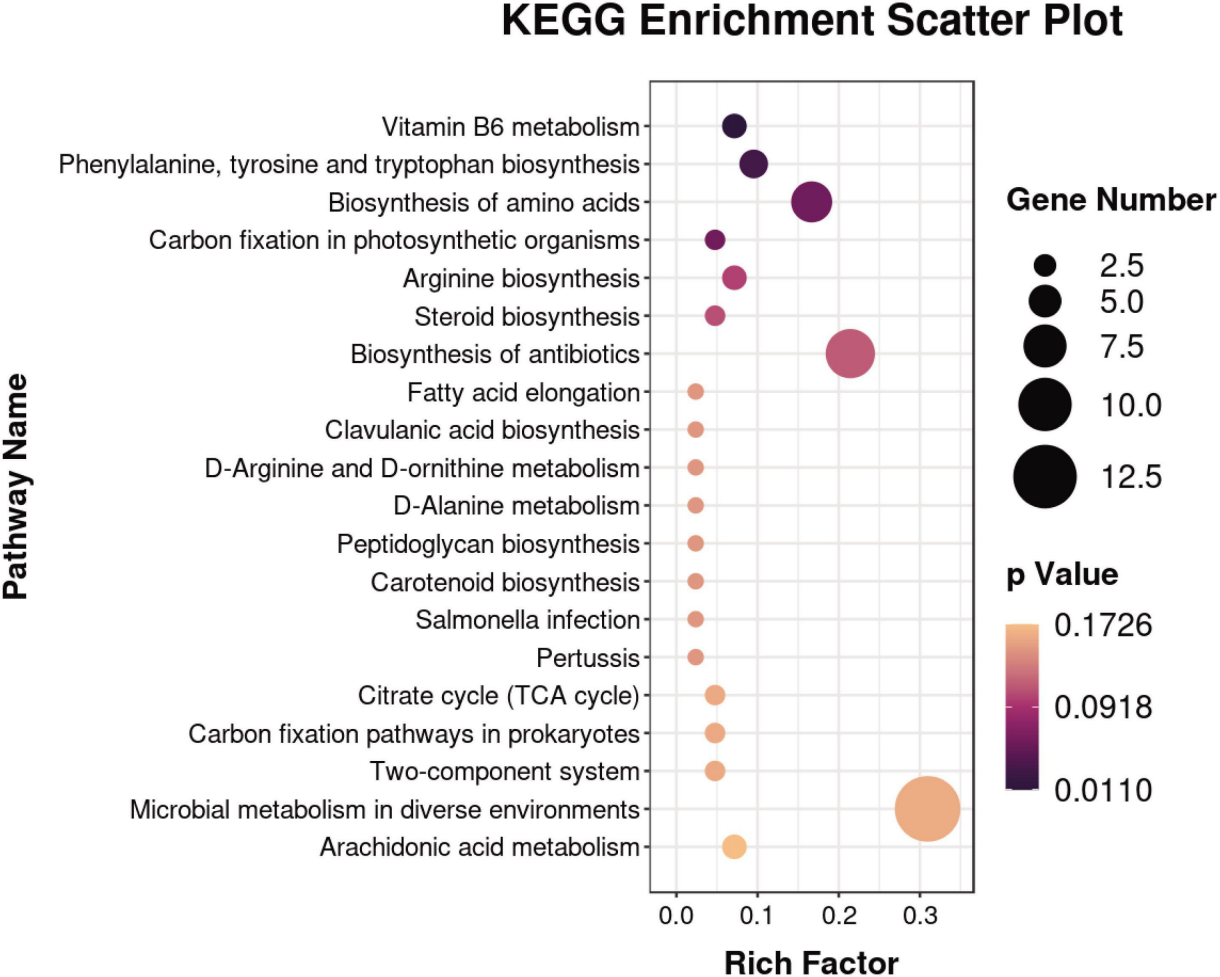
The functions of these metabolites and metabolic pathways were studied using the KEGG database, and a bubble plot displays enriched pathways.

KEGG pathway enrichment analysis is a bioinformatics method for revealing the association of differential metabolites with specific biological pathways in the KEGG database. Pathway enrichment identifies 60 biochemical metabolic and signaling pathways involved in 41 differential metabolites and maps KEGG pathway bubbles (Fig. 6). Four of these pathways are notably associated with the development of pulmonary fibrosis: the biosynthesis of phenylalanine, tyrosine, and tryptophan; arginine biosynthesis; arachidonic acid metabolism; and the pentose phosphate pathway. The data notably indicated significant alterations in vitamin B6 metabolism and the biosynthetic processes of aromatic amino acids (phenylalanine, tyrosine, and tryptophan).

### Microbiota–metabolite interactions in CWP

**Fig 7:**
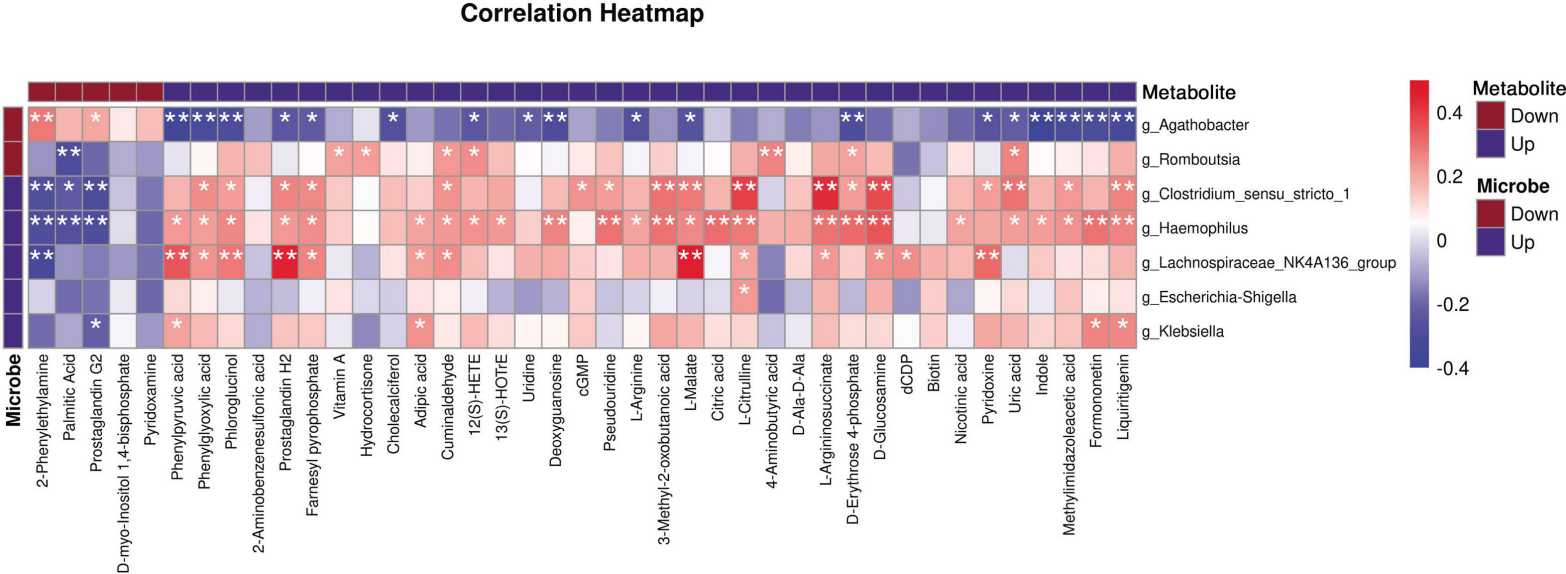
Correlation analysis between the microbiome and metabolome. Spearman’s correlation analysis used differential bacterial genera and 41 differentially abundant metabolites. Red indicates a positive correlation, while blue indicates a negative correlation. ^*^P-value <0.05; **P -value <  0.01.

To gain insight into the interactions between microorganisms and CWP-related metabolites, we selected 7 differential genera and the 41 differential metabolites mentioned above for correlation analysis (Fig. 7). In the context of CWP, we found that bacterial groups with increased abundance were positively correlated with metabolites of increased abundance and negatively correlated with metabolites of decreased abundance. Core bacterial genera, such as *Haemophilus*, *Agathobacter*, *Clostridium_sensu_stricto*_1, and *Lachnospiraceae*_NK4A136_group, were identified with correlations to 28, 20, 20, and 14 metabolites, respectively.

## DISCUSSION

This investigation pioneers the analysis of fecal samples from CWP patients and DEW, employing 16S rRNA high-throughput sequencing and metabolomics to assess alterations in gut microbiota and fecal metabolic profiles in CWP patients. The findings demonstrate notable disparities in gut microbiota composition between the two groups. These shifts in gut microbiota composition may contribute to the deterioration of pulmonary function observed in the early stages of CWP.

This study highlights a significant decline in gut microbiota associated with anti-inflammatory functions and immune regulation in individuals with CWP. Specifically, a noticeable reduction at the phylum level was observed for Actinobacteria and Bacteroidota and at the genus level for *Agathobacter* and *Romboutsia*. Actinobacteria inhibit the growth of pathogenic microorganisms by producing antibiotics, such as penicillin, while activating the host’s immune response and enhancing natural immune function to assist the host in defending itself against pathogenic microorganisms (27, 28). Bacteroidota strengthens the intestinal immune barrier by enhancing IL-36 and macrophage signaling through commensal colonization factor polysaccharide utilization locus (29). *Agathobacter* and *Romboutsia*, known producers of short-chain fatty acids (SCFAs), play critical roles in maintaining gut health and systemic immunity by lowering intestinal pH, enhancing mucin synthesis, and bolstering epithelial integrity (20, 21). This imbalance could reduce the host’s immune capabilities, fostering progressive fibrosis and heightening susceptibility to opportunistic pathogens, potentially accelerating pulmonary fibrosis.

Concurrently, an increase in opportunistic pathogens, such as *Klebsiella*, *Escherichia-Shigella*, *Clostridium_sensu_stricto*_1, and *Haemophilus*, was observed. Notably, *Klebsiella pneumoniae* colonization is facilitated by a disrupted gut microbiome ecology. Reducing Bacteroidota, which normally inhibits such colonization, increases *Klebsiella_pneumoniae* (29). Shigella (including Escherichia coli) is a common opportunistic pathogen, and Shigella infections can stimulate inflammatory responses and are strongly associated with fibrosis in various organs (30–32). Studies have found a correlation between the colonization of Escherichia -Shigella and the promotion of inflammation (33). This process may trigger the production of pro-inflammatory cytokines through an NOD-like receptor family, pyrin domain -containing 3 (NLRP3) -dependent mechanism (34). Increased abundance of Shigella in the gut may lead to decreased immunity, acting as a possible factor in initiating pulmonary fibrosis in CWP patients. Dust exposure in CWP patients correlates with increased *Clostridium_sensu_stricto*_1, a pathogen whose presence in the gut is upregulated by particulate matter exposure (35). In contrast, an increase in *Lachnospiraceae*_NK4A136_group, which is typically anti-inflammatory, was also found (36). In early-stage CWP (stage 1), this increase may represent an adaptive response to slow disease progression (37). Despite these findings, there are still differences in the diversity of the gut microbiota in CWP patients, which may be reflective of variations in the geographical environment. (10, )(27).

The diversity of the gut microbiota and changes in community structure are related to pulmonary function. In HIV-positive individuals with COPD, those with a decrease in FEV1 exhibited markedly lower α -diversity in gut microbiota and distinct flora composition compared to those with normal FEV1 levels (38). Echoing these findings, research in cystic fibrosis patients showed a significant drop in lung microbiota diversity correlating with escalating disease severity, suggesting using microbial diversity combined with pulmonary function for monitoring disease progression (39). Our study aligns with these observations, indicating that compared to CWP patients with normal pulmonary function, CPW patients with impaired pulmonary function have significantly reduced gut microbiota α -diversity and a unique colony structure. No significant microbial differences were detected within the DEW groups based on pulmonary function status. This implies that gut microbial changes may play a role in the decline in pulmonary function in early-stage CWP, supported by correlation analyses between differential microbiota and pulmonary function parameters. A significant inverse relationship was observed between the presence of opportunistic pathogens such as *Klebsiella* and pulmonary function indices. Additionally, studies have suggested that Staphylococcus aureus may reduce pulmonary function by promoting neutrophil proliferation; bacteriophage therapy to eradicate Staphylococcus aureus has shown the potential to improve pulmonary function in emphysematous mice (40). This raises the prospect for further research into the impact of pathogenic bacteria on pulmonary function in CWP and the potential of microbiota-targeted interventions to slow disease progression.

Metabolomics has emerged as a crucial tool for characterizing various levels of metabolites and associated metabolic pathways, offering insights into the underlying mechanisms of diseases. In our study, we conducted a metabolomic analysis of fecal samples from CWP patients and DEW, revealing a disrupted metabolome in individuals with CWP.

Disorders of lipid metabolism have been associated with several lung diseases, including CWP. Our study confirms this association by showing elevated levels of specific membrane phospholipids and lysophosphatidylcholine in CWP patients. In contrast, we observed decreased levels of phosphatidic acid (PA). These are precursor substances of the lung fibrosis mediator lysophosphatidic acid (LPA) (41). This emphasizes the role of the lysophosphatidylcholine-LPA pathway in this disease. The altered PA levels support serum metabolic findings in similar patient studies, highlighting potential avenues for therapeutic intervention (42). Arachidonic acid is critical in pulmonary fibrosis, an essential pathological process in CWP (43). Our findings suggest their involvement in disease progression, indicating elevated levels of the cyclooxygenase pathway metabolite PGH2 and the lipoxygenase pathway metabolite 12(S)-HETE. Interestingly, some arachidonic acid metabolites exacerbate fibrosis, whereas others, such as Eepoxyeicosatrienoic acids, may attenuate fibrosis (44). This dual role highlights the complexity of arachidonic acid metabolism and its potential as a therapeutic target. *Agathobacter* was negatively associated with the discriminative metabolites in this pathway. In contrast, *Haemophilus* displayed a positive correlation with these metabolites, suggesting that *Agathobacter* and *Haemophilus* were collectively responsible for the increased relative abundance of the differentially abundant metabolites in this pathway. Our findings also show an increase in L-carnitine levels in patients with CWP (45, 46) and a decrease in L-carnitine levels in patients with other lung diseases (47), which may indicate a disturbance in the energy metabolism of individuals with CWP.

The study extends to amino acid metabolism, revealing significant increases in L-arginine and its precursors in CWP patients. This could contribute to excessive collagen deposition (48), a hallmark of the disease (49). Another potential factor is the reliance on extracellular arginine due to the absence of arginine synthase in fibroblasts (50). Additionally, elevated levels of aromatic tyrosine and tryptophan and their metabolites indicate an activated immune response (51, 52), adding another layer of complexity to the disease.

Our study revealed an abnormality in the pentose phosphate pathway, evidenced by an increase in D-erythritol-4-phosphate. The pentose phosphate pathway was also disturbed in mice with different triggers of pulmonary fibrosis (53, 54). *Haemophilus*, *Clostridium_sensu_stricto*_1, and *Lachnospiraceae*_NK4A136_group were positively correlated with the pathway metabolites. This may predict that disturbances in the intestinal flora can lead to an imbalance in the body’s antioxidant capacity and promote pulmonary fibrosis in patients with CWP. The significant decline of Ssilibinin in CWP patients and its known therapeutic efficacy make it a potential biomarker for diagnosis and treatment (55).

### Limitations and conclusion

The present investigation highlights a connection between CWP and the gut microbiota. Specifically, certain microbiota, such as Haemophilus and *Clostridium_sensu_stricto*_1, have been identified in association with metabolites known to foster the progression of the disease, implying their potential involvement in the development of chronic pneumoconiosis. However, there are several constraints within the current study. Firstly, it establishes an association between gut microbiota, metabolites, and CWP without elucidating their causal relationship. Thus, a longitudinal cohort study is needed to explore this further. Secondly, despite correction by MaAsLin2, the considerable age difference between subjects in the two groups may introduce confounding factors, necessitating large-scale and meticulously matched studies to validate the results. Thirdly, the absence of a healthy control group not exposed to dust limits the ability to unveil changes in gut microbiota and metabolites from a healthy state to dust exposure and, subsequently, disease development. Fourthly, the study’s comparison was limited to the phenomenological level, underscoring the need for an in-depth exploration of the underlying mechanisms. Lastly, while 16S rRNA sequencing can discern the classification and composition of gut microorganisms, limitations persist in identifying specific species and strains. In conclusion, the study findings indicate significant alterations in the gut microbiota and metabolomic profiles of individuals with CWP. Consequently, further comprehensive investigations across multiple centers are indispensable to unravel the intricate causal mechanisms.

## Data Availability

The raw sequence data reported in this paper have been deposited in the CNCB Data Center: https://ngdc.cncb.ac.cn/gsa (accession no. CRA013357) and https://ngdc.cncb.ac.cn/omix (accession no. OMIX005215).

## SUPPLEMENTAL MATERIAL

Supplemental material spectrum.00049-24-s0001.pdf Tables S1 and S2.

ASM does not own the copyrights to Supplemental Material that may be linked to, or accessed through, an article. The authors have granted ASM a non-exclusive, world-wide license to publish the Supplemental Material files. Please contact the corresponding author directly for reuse.
