# Supplementary material for: Gut microbiome and metabolome profiling in coal workers’ pneumoconiosis: potential links to pulmonary function
Source: Microbiol Spectr. 2024 Sep 16;12(11):e00049-24. doi: 10.1128/spectrum.00049-24 (PMC11537036; doi:10.1128/spectrum.00049-24)
Supplement: Supplemental material — Tables S1 and S2. [file spectrum.00049-24-s0001.pdf]

Table1

Abundant taxa in the gut microbiota of CWP patients and DEW.

Phylum[b]	Family[c]	Genus[d]	CWP	DEW	P value[a]	Q value
Firmicutes			44.90%	38.85%	0.088	
	Lachnospiraceae		19.87%	19.17%	0.827	
		**Agathobacter**	**3.23%**	**4.80%**	**0.011**	**0.031**
		Roseburia	3.32%	2.30%	0.156	
		Blautia	2.03%	2.00%	0.251	
		Fusicatenibacter	0.52%	1.06%	0.265	
		Lachnospira	2.48%	1.74%	0.081	
		Lachnoclostridium	1.83%	2.00%	0.422	
		**Lachnospiraceae_NK4A136_group**	**1.13%**	**0.25%**	**<0.001**	**<0.001**
	**Clostridiaceae**		**1.98%**	**0.51%**	**0.003**	**0.008**
		**Clostridium_sensu_stricto_1**	**1.98%**	**0.51%**	**0.003**	**0.013**
	Ruminococcaceae		10.30%	9.35%	0.294	
		Faecalibacterium	8.16%	7.57%	0.386	
		Succinivibrio	2.61%	4.10%	0.898	
	**Peptostreptococcaceae**		**1.24%**	**1.90%**	**0.004**	**0.011**
		**Romboutsia**	**1.10%**	**1.80%**	**0.006**	**0.022**
	Selenomonadaceae		3.19%	2.51%	0.136	
		Megamonas	3.07%	2.42%	0.131	
	**Veillonellaceae**		**1.40%**	**1.85%**	**0.027**	**0.034**
		Dialister	1.22%	1.29%	0.111	
	**Oscillospiraceae**		**1.50%**	**0.69%**	**0.001**	**0.005**
Bacteroidota			36.68%	46.34%	0.037	

	Prevotellaceae		20.39%	33.05%	0.214	
		Prevotella_9	19.29%	31.57%	0.211	
	Bacteroidaceae		14.73%	11.89%	0.335	
		Bacteroides	14.73%	11.89%	0.308	
Proteobacteria			15.37%	9.51%	0.097	
	**Enterobacteriaceae**		**9.51%**	**2.19%**	**<0.001**	**<0.001**
		**Klebsiella**	**4.63%**	**0.50%**	**<0.001**	**<0.001**
		**Escherichia-Shigella**	**4.53%**	**1.64%**	**0.012**	**0.034**
	Succinivibrionaceae		2.62%	4.10%	0.814	
		Subdoligranulum	1.01%	1.15%	0.914	
	**Pasteurellaceae**		**1.38%**	**0.08%**	**<0.001**	**0.004**
		**Haemophilus**	**1.37%**	**0.08%**	**0.001**	**0.006**
	**Sutterellaceae**		**1.02%**	**1.04%**	**0.044**	**0.047**
Actinobacteria			1.26%	3.35%	0.043	
	Bifidobacteriaceae		1.22%	2.71%	0.185	
		Bifidobacterium	1.22%	2.71%	0.186	

[a]p values are from paired t test. [b]Phylum with average abundance greater than 1% in any of the groups are listed. c. [c]Families with average abundance greater than 1% in any of the groups are listed. [d]Genera with average abundance greater than 1% in any of the groups are listed.

Table2

Differential metabolites identified by untargeted metabolome in this study						
Name	FC	log2FC	Pvalue	ROC	VIP	Up.Down
2-Methoxyestrone	2.270829	1.183219	0.000173	0.744671	1.561221	up
7α-Hydroxytestosterone	2.741539	1.454986	0.000838	0.662306	1.106124	up
12(S)-HETE	2.489669	1.315954	3.97E-06	0.750969	1.098875	up
Hydrocortisone acetate	3.121449	1.642216	0.008923	0.637597	1.099572	up
13(S)-HOTrE	2.757418	1.463318	0.003046	0.640988	1.326708	up
Ecdysterone	2.013046	1.00938	0.024701	0.680233	1.969677	up
T-2 Triol	0.624393	-0.67947	5.35E-06	0.774709	1.078653	down
18-β-Glycyrrhetinic acid	18.04212	4.173297	9.38E-06	0.796512	2.231206	up
2-Aminobenzenesulfonic acid	1.533716	0.617031	0.003588	0.568798	1.241236	up
2-Phenylethylamine	0.650555	-0.62026	1.97E-06	0.796512	1.180626	down
3-Methyl-2-oxobutanoic acid	1.899675	0.925752	0.000333	0.718508	1.046739	up
p-Mentha-1,3,8-triene	1.701942	0.767182	0.002765	0.587209	1.1731	up
Ursolic acid	16.57075	4.050567	0.00662	0.622093	1.210321	up
4-Aminobutyric acid	2.173812	1.120227	0.008104	0.555717	1.039445	up
Sorbitan monostearate	0.538424	-0.89318	2.12E-06	0.807655	1.168741	down
Sorbitan monopalmitate	0.56096	-0.83403	3.84E-06	0.806202	1.102149	down
Adipic acid	2.256808	1.174284	0.00778	0.650194	1.274093	up
2-Methylbutyroylcarnitine	0.567923	-0.81623	4.78E-06	0.757267	1.012992	down
Protectin D1	2.667559	1.41552	7.38E-06	0.76405	2.534432	up
Homo-Gamma-Linolenic Acid (C20:3)	2.091365	1.064445	1.07E-05	0.749031	1.066117	up
11(Z),14(Z),17(Z)-Eicosatrienoic acid	1.561039	0.642506	1.27E-05	0.75436	1.496166	up
Biotin	1.654254	0.726181	0.029919	0.596415	2.308946	up

Prostaglandin E3	1.936332	0.953326	2.42E-05	0.742248	1.02967	up
Bicyclo Prostaglandin E2	2.097634	1.068763	6.32E-05	0.738372	1.454036	up
cGMP	5.586873	2.482041	0.000192	0.693798	1.314601	up
tetranor-PGFM	2.800592	1.485732	8.43E-05	0.738372	1.318756	up
Prostaglandin H1	4.35501	2.122676	0.000109	0.724322	1.209609	up
Prostaglandin K2	6.496875	2.699746	0.000114	0.724806	1.039355	up
12-oxo Phytodienoic Acid	2.994919	1.582517	0.000241	0.695252	1.005767	up
Azelaic acid	1.716743	0.779674	0.000371	0.709787	1.381267	up
2-Isopropylmalate	3.857469	1.947654	0.000796	0.641473	1.014468	up
DL-Panthenol	2.179433	1.123953	0.001304	0.645349	1.006559	up
Ethyl oleate	2.896246	1.534184	0.001941	0.614341	1.146872	up
Cholecalciferol	2.625025	1.392331	0.011276	0.649225	1.085737	up
Oleamide	2.022619	1.016225	0.004133	0.605136	1.411581	up
cis-gondoic acid	2.414775	1.271889	0.007459	0.578973	1.669976	up
Citric acid	4.41983	2.143991	0.020385	0.561047	1.354201	up
Docosatrienoic Acid	0.488426	-1.03379	0.036035	0.627422	2.144208	down
Citramalic acid	2.106649	1.07495	0.042138	0.586725	1.253463	up
8,15-Dihete	2.637434	1.399135	4.18E-06	0.782946	1.61424	up
R-1 Methanandamide phosphate	2.669219	1.416418	0.00027	0.678295	1.63859	up
Cuminaldehyde	4.411818	2.141373	1.13E-05	0.732074	1.034863	up
D-Ala-D-Ala	16.39584	4.035258	5.64E-05	0.667151	1.00235	up
12-Hydroxydodecanoic acid	0.62506	-0.67793	8.11E-06	0.765988	1.028189	down
D-(+)-Malic acid	10.68796	3.417915	0.005029	0.442829	1.18417	up
L-lysine	4.360616	2.124532	3.01E-06	0.766473	1.263945	up
DL-m-Tyrosine	10.65078	3.412887	5.88E-06	0.734981	1.012664	up
Tyrosine	3.750667	1.907147	8.00E-06	0.743702	1.04737	up

N-Acetyl-DL-valine	57.90336	5.855575	9.60E-06	0.75436	1.144394	up
dCDP	2.007725	1.005562	0.00995	0.648256	1.080799	up
Deoxyguanosine	6.391927	2.676251	0.000467	0.703973	1.016424	up
N-Acetylvaline	23.81708	4.573925	3.86E-05	0.704457	1.086576	up
D-Erythrose 4-phosphate	5.628588	2.492773	0.000325	0.656977	1.172868	up
N-acetyl-L-ornithine	8.042255	3.0076	0.000161	0.689438	1.007118	up
Cyclohexaneacetic acid	1.70302	0.768096	0.000317	0.69186	1.036214	up
3-Indoleacrylic acid	8.837873	3.143699	0.000399	0.729651	1.126348	up
D-Glucosamine	9.213406	3.203735	9.77E-06	0.772287	1.176324	up
Tyr-Tyr	2.317465	1.212547	0.002675	0.673934	1.005539	up
Ala-trp	2.905574	1.538823	0.002851	0.655039	1.031182	up
Alanyltyrosine	1.702705	0.767828	0.005531	0.670543	1.082226	up
Ala-Ile	2.737167	1.452683	0.006218	0.677326	1.118568	up
tert-Butyl N-[1-(aminocarbonyl)-3-methylbutyl]carbamate	2.634782	1.397684	0.007019	0.671027	1.220109	up
Gly-Phe	1.964779	0.974367	0.007159	0.656008	1.011721	up
D-myo-Inositol 1,4-bisphosphate	0.522139	-0.93749	0.00401	0.673934	1.497331	down
Cystine	1.5211	0.605115	0.008564	0.48062	1.09843	up
8-Aminooctanoic acid	4.861127	2.281291	0.020228	0.644864	1.751678	up
Farnesyl pyrophosphate	1.814417	0.859506	3.97E-05	0.74564	1.206018	up
Ala-Leu	1.842959	0.882024	0.044489	0.666182	1.220635	up
4-Methyl-2-Oxopentanoic Acid	6.686575	2.741267	6.47E-06	0.791182	1.31103	up
6-Methylquinoline	4.477501	2.162694	0.003488	0.657461	1.180036	up
Methyl nicotinate	2.45121	1.293494	2.82E-06	0.79312	1.081217	up
Formononetin	3.493044	1.804485	0.044123	0.662791	1.182391	up
Hydrocortisone	3.072106	1.619228	0.007246	0.481589	1.246604	up
Indole	3.387596	1.760262	0.005635	0.665698	1.311121	up

NSI-189	1.932781	0.950678	0.000668	0.709302	1.032389	up
Tetrahydroaldosterone	2.275136	1.185953	1.65E-06	0.770349	2.24094	up
5-Hydroxyindole	3.996007	1.998559	7.08E-06	0.803779	1.314444	up
Indole-3-carbinol	3.314166	1.728646	8.23E-06	0.756783	1.143621	up
Skatole	3.907126	1.966108	2.19E-05	0.797965	2.507241	up
L-Arginine	5.560343	2.475174	0.000706	0.690891	1.02313	up
DL-Tryptophan	4.583991	2.196604	0.011633	0.647287	1.407641	up
trans-3-Indoleacrylic acid	4.60208	2.202286	0.013626	0.646802	1.436871	up
L-Argininosuccinate	3.059633	1.613359	1.83E-05	0.732074	1.051056	up
6-(Dimethylamino)purine	2.354552	1.235452	0.001555	0.685562	1.037078	up
L-Citrulline	62.2863	5.960843	1.36E-05	0.769864	1.075482	up
Liquiritigenin	9.614698	3.265242	2.91E-05	0.785853	2.226628	up
Sunitinib	1.624587	0.700073	0.02099	0.500969	1.612035	up
Paracetamol	14.35306	3.843287	2.29E-05	0.781008	1.620049	up
L-Malate	2.101311	1.07129	2.92E-06	0.785368	1.399418	up
4-Butylresorcinol	1.600648	0.678656	2.91E-05	0.733527	1.104601	up
2-Naphthol	2.620408	1.389792	1.23E-07	0.809593	2.253008	up
Methylimidazoleacetic acid	18.31607	4.195038	9.10E-06	0.725775	1.01779	up
Nicotinic acid	16.9587	4.083954	0.000129	0.690891	1.122422	up
Palmitic Acid	0.609045	-0.71538	7.89E-05	0.729167	1.444203	down
3,4-Dimethylbenzoic acid	2.71411	1.440479	0.000258	0.70688	1.29443	up
2-Hydroxyhippuric acid	10.33592	3.369595	0.001105	0.693798	1.527155	up
Phenylglyoxylic acid	2.634456	1.397505	5.68E-06	0.754845	1.044172	up
4-Toluic acid	1.855634	0.891912	0.00427	0.694283	1.163083	up
2,5-Dimethylphenol	7.67237	2.939672	0.010864	0.639535	1.168528	up
4-(Diethylamino)benzaldehyde	2.610899	1.384547	4.83E-05	0.671512	1.152263	up

2-(3,4-dimethoxyphenyl)ethanamine	2.897211	1.534665	0.000371	0.632267	1.187044	up
IPH	8.367249	3.064753	0.000819	0.687016	1.113542	up
Epinephrine bitartrate	5.740361	2.521142	2.39E-06	0.790213	1.256273	up
LPI 18:0	2.201304	1.138358	1.78E-05	0.752422	4.477207	up
LPG 15:0	1.744547	0.802853	4.65E-05	0.728682	1.288152	up
LPE 19:1	2.525758	1.336717	5.75E-05	0.751453	2.218474	up
PE (14:0/15:0)	4.284729	2.099204	5.87E-05	0.750484	1.152276	up
PE (17:1/17:1)	6.222615	2.637521	0.000227	0.664244	1.282559	up
LPG 18:0	1.504894	0.589661	0.000424	0.765019	1.926644	up
LPE 17:1	3.692758	1.884699	0.002356	0.674903	1.819164	up
Lysope 14:0	2.205003	1.140781	0.00292	0.618702	1.092923	up
LPS 15:1	1.817049	0.861597	0.003562	0.713178	1.050951	up
Lysopc 20:0	1.626385	0.701669	0.03502	0.594477	1.307461	up
LPS 22:0	2.209621	1.143799	0.036071	0.678779	1.962059	up
PA (12:0/12:0)	0.628648	-0.66968	1.16E-05	0.717539	1.011833	down
6-Deoxy-D-glucose	0.610079	-0.71293	5.83E-06	0.747578	1.006789	down
Phenylpyruvic acid	2.276783	1.186997	6.14E-05	0.756298	1.190185	up
Dl-3-Hydroxy-kynurenine	1.889912	0.918319	0.001744	0.547481	1.109948	up
Phloroglucinol	1.639753	0.713478	2.67E-05	0.74031	1.060301	up
Prostaglandin G2	0.496902	-1.00897	9.63E-07	0.768895	1.726535	down
Prostaglandin H2	2.272072	1.184009	1.42E-05	0.767926	1.25754	up
AMK	1.75834	0.814214	0.01563	0.661822	1.001394	up
Pseudouridine	3.458793	1.790269	0.007849	0.592539	1.234269	up
Pyridoxamine	0.565531	-0.82232	5.61E-05	0.761143	1.202513	down
Pyridoxine	17.01126	4.088418	1.32E-05	0.76405	1.583383	up
1-Methyladenosine	3.478096	1.798298	2.50E-06	0.748062	1.109163	up

Uric acid	8.444116	3.077946	0.000275	0.755814	1.052707	up
S-(5-Adenosy)-L-Homocysteine	1.969689	0.977968	9.83E-06	0.782461	1.38394	up
DL-4-Hydroxyphenyllactic acid	2.689587	1.427385	6.86E-05	0.74564	1.104369	up
3-(4-Hydroxyphenyl)propionic acid	2.943528	1.557547	0.000994	0.656492	2.113784	up
3-(3-Hydroxyphenyl)propionic acid	6.539782	2.709243	0.019128	0.628876	1.101574	up
Uridine	2.575866	1.365057	0.030233	0.626453	1.162783	up
Vitamin A	1.891172	0.91928	2.03E-05	0.710271	1.09551	up
Normorphine	19.67054	4.297965	0.002365	0.616279	1.043788	up
1-Palmitoylglycerol	1.836673	0.877095	0.000105	0.739826	1.097311	up
5 methyl THF	1.603736	0.681437	3.45E-05	0.665213	1.125333	up
Silibinin	0.212935	-2.23151	0.003191	0.827035	1.367951	down
L-Carnitine	1.74719	0.805036	0.001712	0.575581	1.123046	up
3-(4-hydroxyphenyl)propanohydrazide	1.97113	0.979023	7.45E-08	0.8125	2.113734	up
2-[2-oxo-2-(2-pyridylamino)ethoxy]acetic acid	3.800878	1.926333	1.80E-06	0.786337	1.471699	up
(±)9(10)-DiHOME	0.54767	-0.86862	2.94E-06	0.82219	1.146597	down
2-(2-oxo-2-{[2-(2-oxo-1-imidazolidinyl)ethyl]amino}ethoxy)acetic acid	39.24773	5.294537	3.23E-06	0.733527	1.101827	up
2-methyl-2,3,4,5-tetrahydro-1,5-benzoxazepin-4-one	1.949079	0.962792	3.25E-06	0.764535	1.567896	up
2-{[(6-fluoro-4H-1,3-benzodioxin-8-yl)methyl]thio}-1,3-benzothiazole	13.93699	3.800848	3.80E-06	0.739341	1.039233	up
FIBF-d7	0.652913	-0.61504	4.72E-06	0.752422	1.120863	down
1-(3-chloro-4-methylphenyl)-2-[(4-chlorophenyl)sulfonyl]propan-1-one	3.938595	1.977681	5.38E-06	0.729651	1.115945	up
N-(4-piperidinophenyl)-2-thiophenecarboxamide	33.79737	5.078839	6.09E-06	0.74564	1.033814	up
TNH	7.088053	2.825389	6.41E-06	0.732074	1.110613	up
TMK	40.0454	5.323565	7.54E-06	0.745155	1.005119	up
N1-tetrahydrofuran-2-ylmethyl-2-cyanoacetamide	0.61051	-0.71191	7.57E-06	0.763081	1.004798	down
Chenodeoxycholic acid-3-beta-D-glucuronide	2.157987	1.109686	9.90E-06	0.775194	2.145743	up
2-(2-carboxy-2-methylpropyl)-4,6-dimethylbenzoic acid	2.188675	1.130058	1.43E-05	0.750969	1.251954	up

N'-(cyclohexylcarbonyl)-4-methyl-1,2,3-thiadiazole-5-carbohydrazide	2.632393	1.396375	1.46E-05	0.749516	1.348921	up
SLH	41.55305	5.376883	1.54E-05	0.733043	1.014803	up
N-(2,5-diethoxy-4-morpholinophenyl)acetamide	6.45695	2.690853	1.72E-05	0.721415	1.032703	up
4,7-dimethylpyrazolo[5,1-c][1,2,4]triazine-3-carbonitrile	3.13837	1.650016	1.95E-05	0.719961	1.012397	up
N1-{2-[(2,2-dicyanovinyl)amino]-4-fluorophenyl}acetamide	2.149335	1.10389	2.06E-05	0.761143	2.120207	up
N~5~-(1,3,5-trimethyl-1H-pyrazol-4-yl)-1H-1,2,4-triazole-3,5-diamine	2.939514	1.555578	2.07E-05	0.732558	1.123511	up
Ip7G	0.578144	-0.7905	2.75E-05	0.743702	1.074765	down
DPK	3.662095	1.872669	3.68E-05	0.712209	1.108508	up
HBMP (12:0-18:0-20:0)	0.636874	-0.65092	3.91E-05	0.748547	1.038642	down
SPK	3.7569	1.909543	4.37E-05	0.732558	1.091545	up
tetranor-12(R)-HETE	3.803615	1.927371	4.91E-05	0.757752	1.46225	up
2-[6-(1H-benzo[d]imidazol-2-yl)-2-pyridyl]-1H-benzo[d]imidazole	8.709738	3.122629	5.34E-05	0.823159	2.303583	up
4-Pregnen-17alpha,20alpha-Diol-3-One	0.538533	-0.89289	5.49E-05	0.728682	1.511276	down
Lysopc 16:2 (2N Isomer)	1.66248	0.733337	0.000106	0.73062	1.412002	up
[1,1'-biphenyl]-2,2'-dicarboxylic acid	1.74129	0.800156	0.000183	0.79312	3.077904	up
N1-[2-(4-chlorophenyl)-2-oxoethyl]-4-chlorobenzamide	3.752442	1.90783	0.000218	0.639535	1.063669	up
N-(4-butyl-2-methylphenyl)-N'-hydroxyiminoformamide	2.451194	1.293485	0.000244	0.707364	1.234909	up
2-(2-chlorophenyl)-1-cyclohexyl-6-oxopiperidine-3-carboxylic acid	3.254816	1.702576	0.000249	0.752907	1.43681	up
diethyl 3-amino-6-methylthieno[2,3-b]pyridine-2,5-dicarboxylate	3.358024	1.747613	0.000272	0.765988	2.43988	up
N4-(4-chloro-2,5-dimethoxyphenyl)morpholine-4-carbothioamide	3.482479	1.800115	0.000393	0.617248	1.04162	up
(2R,3S,4S,5R,6R)-2-(hydroxymethyl)-6-(propan-2-yloxy)oxane-3,4,5-triol	6.547705	2.710989	0.000402	0.670543	1.005623	up
YNK	2.078411	1.055481	0.00052	0.693798	1.007058	up
(±)11(12)-DiHET	3.069686	1.618091	0.000542	0.662791	1.056365	up
3,5-di(2-furylmethylidene)tetrahydro-2H-pyran-4-one	3.128792	1.645606	0.000625	0.676841	1.479453	up
Noroxycodone-d3	3.062437	1.61468	0.000733	0.636628	1.092562	up
5,7-dimethyl-2-phenylpyrazolo[1,5-a]pyrimidine	8.54426	3.094956	0.000753	0.563953	1.270773	up

FQH	4.560878	2.189312	0.000782	0.686047	1.063283	up
(3S,9aS)-3-benzyl-octahydro-1H-pyrido[1,2-a]pyrazin-1-one	2.024492	1.01756	0.00084	0.663275	1.042023	up
ethyl 1-(3-nitro-2-thienyl)piperidine-4-carboxylate	36.36967	5.184664	0.000863	0.704942	1.001354	up
3-(2-naphthyl)-5-(trifluoromethyl)-1H-pyrazole	42.37406	5.405109	0.000864	0.713178	1.471591	up
KMH	3.466313	1.793402	0.000867	0.673934	1.012807	up
3-[3-(beta-D-Glucopyranosyloxy)-2-hydroxyphenyl]propanoic acid	7.417266	2.890887	0.000984	0.719477	1.129833	up
3-(pyrazin-2-ylamino)-2-(2-thienylcarbonyl)acrylonitrile	2.067639	1.047984	0.001236	0.757267	1.352095	up
methyl 2-(6-hydroxy-3-oxo-3H-xanthen-9-yl)benzoate	4.931622	2.302062	0.001241	0.63469	1.032057	up
2-[2-oxo-2-(pyridin-3-ylamino)ethoxy]acetic acid	2.087387	1.061698	0.001397	0.679264	1.175919	up
1,6-Hexanediol diacrylate	2.940609	1.556115	0.001406	0.651647	1.319178	up
4-[2-(2-oxo-1-imidazolidinyl)ethyl]-1lambda~6~,4-thiazinane-1,1-dione	4.07814	2.027911	0.001639	0.651163	1.025248	up
DMK	2.1844	1.127237	0.001815	0.669089	1.330444	up
(2-anilino-4-methyl-1,3-thiazol-5-yl)(4-methoxyphenyl)methanone	1.789082	0.83922	0.002691	0.71657	1.075769	up
EPK	3.320164	1.731255	0.002928	0.653585	1.145681	up
MMK	2.105584	1.07422	0.003026	0.662791	1.101677	up
ethyl 5-methoxy-2-methyl-1-phenyl-1H-indole-3-carboxylate	2.339626	1.226278	0.003078	0.629845	1.14272	up
1,6-dihydroxy-3-methoxy-8-methyl-9H-xanthen-9-one	0.638062	-0.64823	0.004124	0.666182	2.631167	down
6-cyclohex-3-enyl-5-nitropiperidin-2-one	5.846222	2.547505	0.004245	0.608043	1.108594	up
2-methyl-4-[(3-methyl-2-thienyl)methylene]-1,3-oxazol-5(4H)-one	2.397218	1.261361	0.004299	0.664729	1.154426	up
(12Z)-9,10,11-trihydroxyoctadec-12-enoic acid	1.970576	0.978617	0.00483	0.587694	1.250305	up
2-[(butylamino)(imino)methyl]-1-oxohydrazinium-1-olate	1.751267	0.808399	0.004957	0.640504	1.050627	up
N-(2,5-diethoxy-4-morpholinophenyl)-4-methoxybenzenesulfonamide	1.882229	0.912442	0.005622	0.630814	1.475955	up
2-oxopiperidine-3-carbohydrazide	1.508693	0.593299	0.006056	0.673934	1.136016	up
GNK	1.820045	0.863974	0.006084	0.650194	1.216664	up
12,13-EODE	2.895789	1.533957	0.007621	0.607558	1.731092	up
4-phenoxyphenyl 4-hydroxypiperidine-1-carboxylate	7.969335	2.994459	0.00914	0.592539	1.176609	up

2-{[(4-phenyl-1H-pyrazol-5-yl)amino]methylene}-1H-indene-1,3(2H)-dione	5.390843	2.430511	0.011415	0.624031	1.142366	up
3-pentadecyl-4,5,6,7-tetrahydrobenzo[d]isoxazol-4-one oxime	2.622286	1.390825	0.012161	0.648256	1.139248	up
RMH	1.889547	0.918041	0.012849	0.628876	1.091387	up
5,6-dimethyl-4-oxo-4H-pyran-2-carboxylic acid	1.864769	0.898997	0.013384	0.620155	1.050449	up
6,8-dihydroxy-3-(10-hydroxyundecyl)-3,4-dihydro-1H-2-benzopyran-1-one	2.506057	1.325419	0.014005	0.593508	1.208088	up
ILK	2.124005	1.086787	0.015939	0.652616	1.129599	up
QLH	1.584746	0.664252	0.018827	0.640504	1.000254	up
PNK	5.822908	2.54174	0.020796	0.609981	1.131517	up
2-[(carboxymethyl)(methyl)amino]-5-methoxybenzoic acid	1.969145	0.977569	0.026341	0.632752	1.055533	up
ENK	1.508503	0.593117	0.030215	0.612403	1.171773	up
GLK	1.691547	0.758343	0.031668	0.631298	1.10032	up
EKK	2.352239	1.234034	0.03413	0.624516	1.137247	up
DLK	1.618143	0.694339	0.042269	0.627907	1.361047	up
3-(2,3-dihydro-1H-indol-1-yl)-2-[(2-furylmethyl)sulfonyl]acrylonitrile	5.274684	2.399085	0.046735	0.45688	1.084636	up
